# Do Facial Expressions Develop before Birth?

**DOI:** 10.1371/journal.pone.0024081

**Published:** 2011-08-31

**Authors:** Nadja Reissland, Brian Francis, James Mason, Karen Lincoln

**Affiliations:** 1 Department of Psychology, University of Durham, Durham, United Kingdom; 2 Department of Mathematics and Statistics, Lancaster University, Lancaster, United Kingdom; 3 Wolfson Research Institute, University of Durham, Stockton, United Kingdom; 4 The James Cook University Hospital, Middlesbrough, United Kingdom; McGill University Health Center, Canada

## Abstract

**Background:**

Fetal facial development is essential not only for postnatal bonding between parents and child, but also theoretically for the study of the origins of affect. However, how such movements become coordinated is poorly understood. 4-D ultrasound visualisation allows an objective coding of fetal facial movements.

**Methodology/Findings:**

Based on research using facial muscle movements to code recognisable facial expressions in adults and adapted for infants, we defined two distinct fetal facial movements, namely “cry-face-gestalt” and “laughter- gestalt,” both made up of up to 7 distinct facial movements. In this conceptual study, two healthy fetuses were then scanned at different gestational ages in the second and third trimester. We observed that the number and complexity of simultaneous movements increased with gestational age. Thus, between 24 and 35 weeks the mean number of co-occurrences of 3 or more facial movements increased from 7% to 69%. Recognisable facial expressions were also observed to develop. Between 24 and 35 weeks the number of co-occurrences of 3 or more movements making up a “cry-face gestalt” facial movement increased from 0% to 42%. Similarly the number of co-occurrences of 3 or more facial movements combining to a “laughter-face gestalt” increased from 0% to 35%. These changes over age were all highly significant.

**Significance:**

This research provides the first evidence of developmental progression from individual unrelated facial movements toward fetal facial gestalts. We propose that there is considerable potential of this method for assessing fetal development: Subsequent discrimination of normal and abnormal fetal facial development might identify health problems in utero.

## Introduction

The development of complex facial movements in the uterus is essential for a number of functions after birth, such as the infant sucking movement involved in feeding from breast or bottle [1], jaw and tongue movements necessary for speech [2] and movements in the face necessary for facial expressions. Hence, fetal motor development affects not only general motor and specific oral-motor function in neonates [3] but also but also their facial expressions.

Evaluation of fetal facial expressions may be a key to predicting the healthy brain function of the fetus [4] which can be observed behaviourally from the late embryonic period onwards. Although the innervations of well-formed facial muscles begin very early in gestational age at about 8 weeks [5] and by 16 weeks all of the muscles used in facial expressions are formed [6], it is only later, between the gestational ages of 24 to 36 weeks that the deposition of facial adipose tissue gradually builds up. During this period it may be possible to assess age–related developments of fetal facial movements by adapting the coding systems already in use for newborn and premature babies [7], [8] and applying such a scheme on modern 4-D ultrasound recordings (depth-enhanced movie imaging).

Evidence for the potential of this approach comes from in-utero descriptive observation studies. As the fetus develops, fetal movements of the face, limbs and torso are correlated with the structural development of the central nervous system [9], demonstrating that the connection between cerebral cortex and peripheral structures of the fetus are functional. In addition to simple muscle movements, cerebral cortex functions are developed to facilitate learning in the fetus prenatally [9]. For example, fetuses in the last weeks of gestation can already show habituation, memory and reaction to sounds [10]–[12]. In compromised fetuses, development progresses with gestation, although progress is at variance with that in healthy fetuses, indicating that fetal facial movements may serve as a marker for normal development [13]–[15].

At present, comparisons of study findings are limited because of the use of fundamentally different recording and coding conventions. Although facial movements, controlled by V and VII cranial nerves, appear around 10 and 11 weeks [16], the exact onset of facial expressions has not been determined and it is still unclear whether their appearance is related to gestational age. For example, studies have reported variously “grimacing” in the age range from 7–40 weeks gestation [17]; perceived “smiling” and “grimacing” at 33 weeks [18]; eye blink and mouth movements at 20 to 38 weeks [19] and cry movement assessed by 2-D scans imaging bone mass rather than muscle movement at 33 weeks [20]. A study of 10 fetuses, with a median gestational age of 30 weeks, using 4-D ultrasound, observed ‘smiling’ once or more in 8 out of the 10 fetuses and ‘scowling’ in 6 of the 10 fetuses [4]. One group found a decrease in facial expressions with increasing gestational age, although this may be an artefact of the method employed [17]. Heterogeneity of method and coding within studies has dramatically limited advances in understanding.

The Facial Action Coding System (FACS) for infants [8], [21] and for adults [22] and classifies all observable facial muscle movements promoting objective assessment, and, unlike other schemes which relate specifically to emotion faces such as pain[7], is emotion-free. Although, using 4D imaging, some selected fetal facial movements have been described in recent years, there is, as yet, no comprehensive coding system of such facial movements applied to fetuses. Crucially we lack insight into how fetal facial movements become coordinated over time to form recognisable emotional expressions or ‘gestalts’ such as the cry face gestalt [23]. The aim of the present study was to develop a reliable and reproducible method for fetal facial movement coding.

## Methods

### Objectives

The present study was designed to address two questions. Does the complexity of facial movement increase with fetal age? If so, do facial gestalts, one showing facial movements associated with positive and the other with negative emotions in babies and children (e.g. “laughter” gestalt or “cry-face”-gestalt) emerge from the second to third trimester of pregnancy? These hypotheses were explored with two healthy fetuses using frame by frame coding of facial actions in order to develop a reliable and reproducible method for fetal facial movement coding.

### Participants

Two healthy fetuses, both girls, were scanned. Mothers were specifically recruited for this study through the midwives of the antenatal unit of the James Cook University Hospital following ethical procedures. The first fetus, a girl, was observed at 24, 27 and 34 weeks and was 9 pounds 8 ounces at birth and the second, also a girl, at 24, 28, 32 and 35 weeks gestational age and was 7 pounds 4 ounces at birth, with Apgar scores of 9 at one and five minutes after birth, indicating both were healthy full-term babies.

### Procedure

Following ethical guidelines mothers were scanned on each occasion for 20 minutes. Mothers were made aware before the procedure that these additional scans were not performed for medical reasons but in order to establish the range of facial movements a fetus can show at various gestational ages. After mothers had received their 20 week anomaly scan in which their fetus was found to be normal and where no data on facial movements were collected, both mothers received additional scans in which the fetuses while they were active were observed for approximately 20 minutes on each occasion. The fetal face and upper torso were visualized by means of 4D full frontal or facial profile ultrasound recordings, and recorded for off line analysis with a GE Voluson 730 Expert Ultrasound System using a GE RAB4–8L Macro 4D Convex Array Transducer.

For each observation period, we coded 600 seconds of scan (which were not necessarily consecutive) when the full face was visible; starting with the first moment when the full face was codable. No stimulation was applied in these observation periods.

### Method of coding

The Facial Action Coding System [22] is an anatomically based system that itemises facial muscle movements, or “action units” (AUs). Using a method designed for the eye brow region of the face [24] which defined movements in the upper face in relation to FACS [22] as well as the web resource Artnatomy [25], we identified 19 fetal facial movements which could be observed and reliably coded from fetal 4 D scans. These 19 movements identified are based on definitions of FACS [22] and BabyFacs [8], [21], [26] (see Table 1 column 1). We translated movement combinations into specific “gestalts” by identifying facial configurations which are classified as “cry” and “laughter” gestalts in Artnatomy [25]. Although Table 1 identifies that the cry and laughter gestalts have a number of AUs in common, both the cry and laughter gestalts have two unique AUs which separate them. However, in terms of defining a full cry or laughter gestalt, we specified that all AUs were equally important. We tried to avoid value judgements by naming the configuration of facial movements necessary in order to show a “cry-gestalt” and “laughter-gestalt” rather than calling these gestalts “positive” or “negative” expressions. Facial movements were coded using a modified list of action units (AUs) from the Facial scheme. We assessed reliability of the coding by independently re-coding twenty percent of recordings for fetus A. Using Cohen's Kappa, the overall reliability of coding at 24, 27 and 34 weeks was .87, .94 and .94; for the cry face gestalt was .91, .86 and .94; and, for the laughter face gestalt was .93, .91 and .94.

**Table 1:** The 19 action units of the coding scheme and the definition of the cry-face and laughter-face gestalts based on this scheme indicated by tickmarks.

The 19 action units	Cry – face gestalt	Laughter face gestalt
1. Inner Brow Raise (AU1)	<$>\raster="rg1"<$>	
2. Outer Brow Raise (AU2)		
3. Brow Lowerer (AU4)	<$>\raster="rg1"<$>	<$>\raster="rg1"<$>
4. Cheek Raiser (AU 6)	<$>\raster="rg1"<$>	<$>\raster="rg1"<$>
5. Nose Wrinkle(AU9)	<$>\raster="rg1"<$>	<$>\raster="rg1"<$>
6. Upper Lip Raiser (AU 10)		
7. Nasolabial Crease (AU11)	<$>\raster="rg1"<$>	<$>\raster="rg1"<$>
8. Lip Pull (Au12)		<$>\raster="rg1"<$>
9. Dimpler (AU14)		
10. Lip Corner Depressor (AU15)		
11. Lower Lip Depressor (AU 16)	<$>\raster="rg1"<$>	
12. Chin Raiser (AU17)		
13. Lip Pucker (AU 18)		
14. Tongue Show (AU19)		<$>\raster="rg1"<$>
15. Lip Stretch (AU20)		
16. Lip Presser (AU 24)		
17. Lip parting (AU25)	<$>\raster="rg1"<$>	<$>\raster="rg1"<$>
18. Mouth Stretch (AU27)	<$>\raster="rg1"<$>	<$>\raster="rg1"<$>
19. Lip Suck (AU28).		

Note: For both cry-face and laughter gestalts, either AU25 orAU27 can occur, but not both together.

### Ethics

Ethical permission was granted by the County Durham and Tees Valley 2 Research Ethics Committee (REC Ref: 08/H0908/31), James Cook University Hospital. Both mothers gave informed written consent.

### Statistical methods

Co-occurring facial movements were those within 1 second of one another. Specific AUs were assigned to two gestalts ‘cry’ and ‘laughter’ (see Table 1 columns 2 and 3). It should be noted that the two gestalts are similar on a number of the AUs, but differ according to AU1, AU12, AU16, and AU19. However, the full gestalt needs to be defined using both common AUs and unique AUs. Events were coded according to the number of AUs observed, for example single, double, triple events and so on, and were then aggregated for each observation period to produce a set of counts identifying the number of single, double, triple etc events.

Ordinal regression [27] was then used to assess whether there was a tendency for fetal movements to become more complex over time – both generally and within specific gestalts. This permitted a test for association between the co-occurrence of AUs and linear age (on the cumulative log-odds scale). We analysed each fetus separately, as we expected that fetuses would show variability in behaviour. Using a proportional odds model for the ordinal data [28], two models were fitted to the observed counts for each fetus - one with linear age as a covariate, and one without. Where necessary, we also tried using the exponent of (age - 24 weeks) as an alternative covariate, which allowed for age having a non-linear and increasing effect for higher gestational ages. Significance was determined through a likelihood ratio test (LRT) of the two models. Goodness of fit of the linear model was also assessed through the model (X^2^) Pearson chi-squared test [27]. All analyses were carried out using SPSS for Windows [29].

## Results

**Figure 1:**
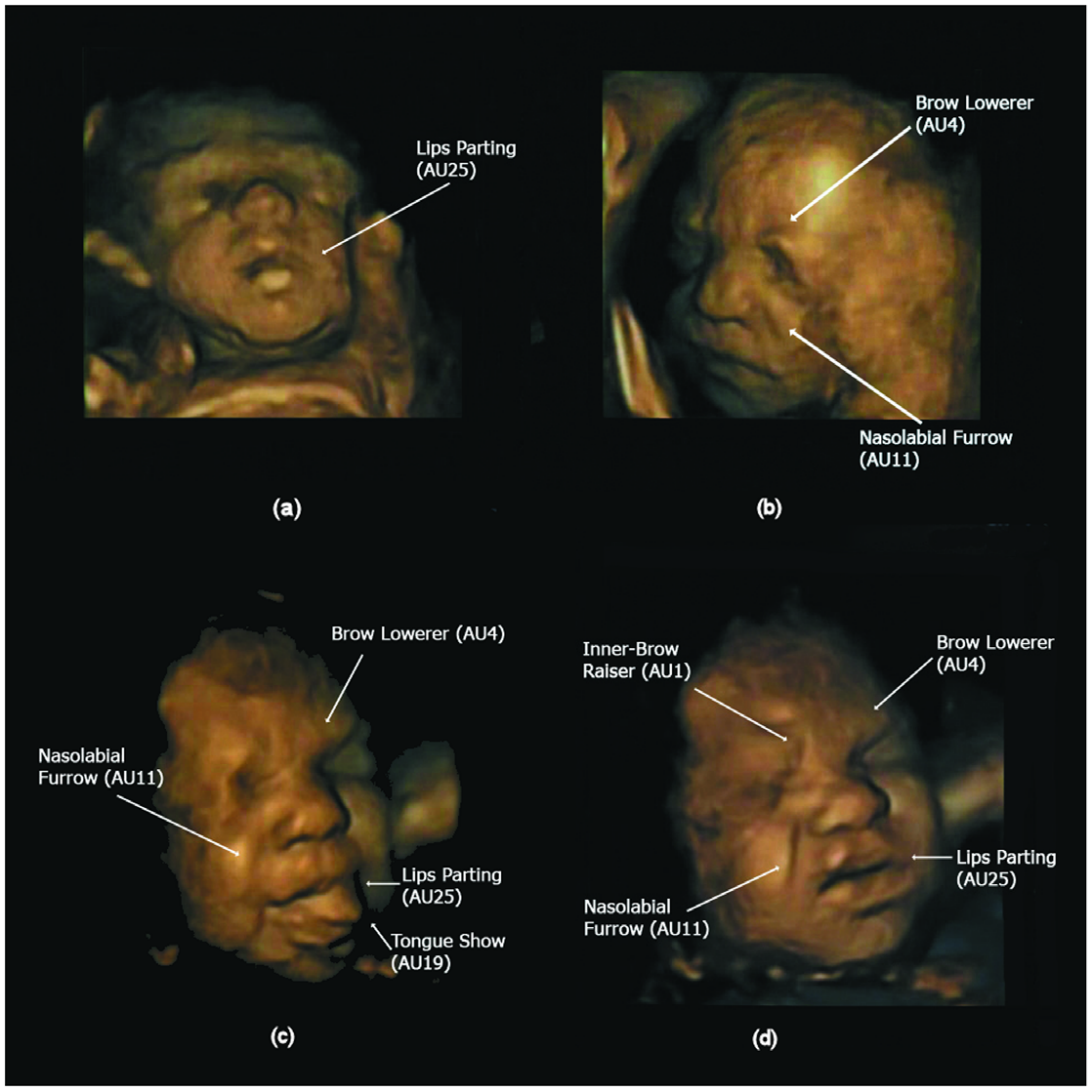
Illustration of developmental progression in number of action units (AUs) over gestational age. (a) One AU at 24 weeks (b) Two AUs at 27.5 weeks (c) and (d) two different combinations of 4 AUs at 32 weeks.

**Figure 2:**
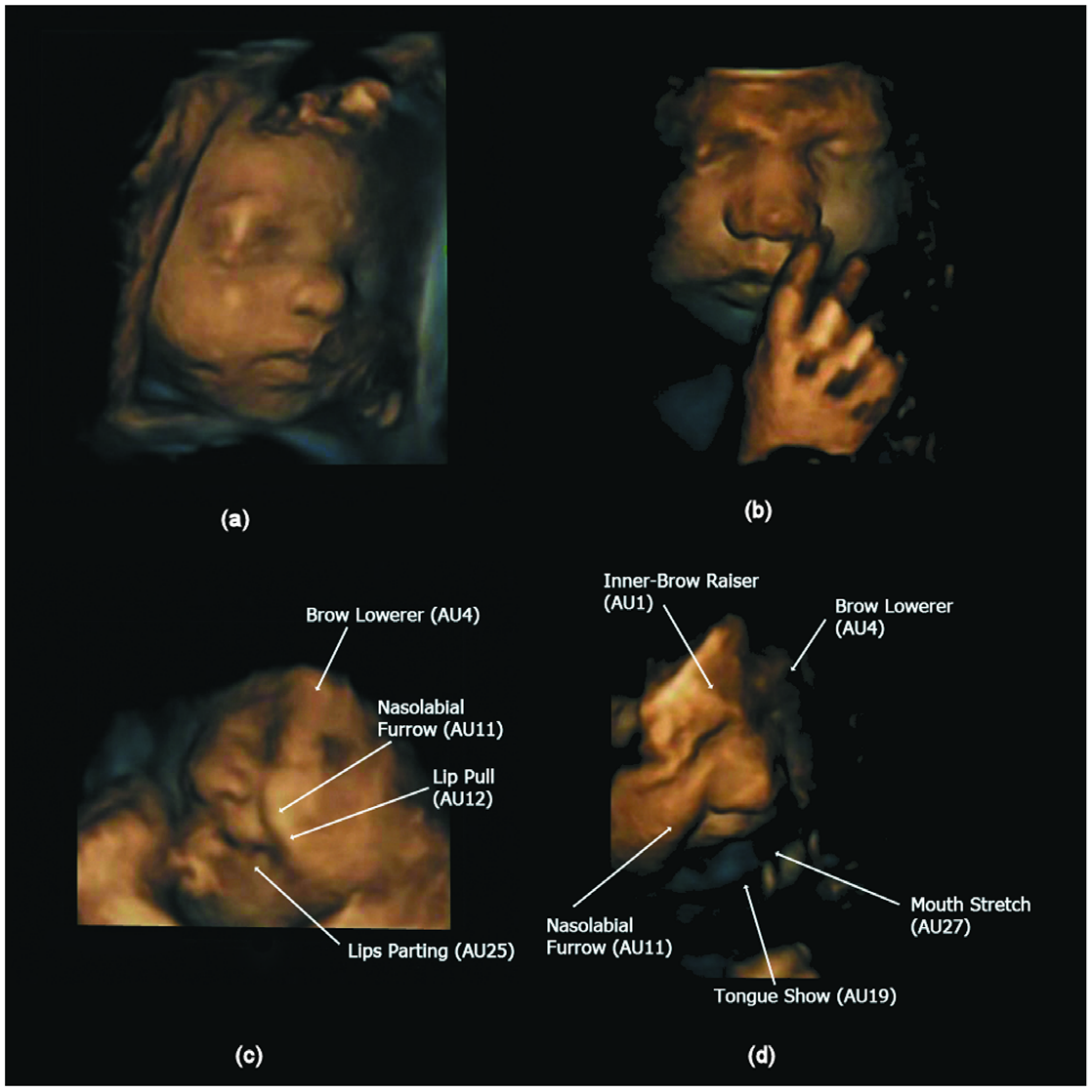
Illustration of neutral faces over gestational age and combinations of action units (AUs) for the laughter and cry gestalts. (a) neutral face at 28 weeks and (b) at 33 weeks. (c) 4 AUs contributing to the laughter gestalt at 32.5 weeks and d) 5 AUs contributing to the cry gestalt at 33 weeks.

Table 2 reports the observed total number of single, double, triple etc facial events (AUs) over the two fetuses combined. It also reports on the total number of events observed within each gestalt. The pattern of co-occurrence of all facial events increased with fetal age (Table 2). In addition, the co-occurrence of facial events contributing to cry and laughter gestalts also increased with age. Figure 1 depicts the developmental progression of AU combinations with increasing gestational age showing (a) one AUs at 24 weeks (b) two AUs at 27.5 weeks, and (c,d) two different instances of four AUs at 32 weeks. We were able to observe events with up to seven AUs co-occurring. An ordinal regression therefore used a six-category ordinal variable as response (single AU, double, triple, quadruple, quintuple and sextuple or more). Figure 2 illustrates two neutral faces with no AUs present (a) at 28 weeks and (b) at 33 weeks, as well as combinations of AUs involved in (c) the laughter gestalt at 32.5 weeks and (d) the cry gestalt at 33 weeks.

**Table 2:** Co-occurrence of action units (AUs) across the two fetuses by selected fetal ages.

	Single	Double	Triple	Quadruple	Quintuple	Sextuple	Total
**All AUs**							
24	81	47	9	2	0	0	139
weeks	(58%)	(34%)	(6%)	(1%)			
27–28	57	63	13	3	0	0	136
weeks	(42%)	(46%)	(10%)	(2%)			
34–35	17	37	55	39	15	6	169
weeks	(10%)	(22%)	(33%)	(23%)	(9%)	(4%)	
**Cry Face AUs**							
24weeks	75 (97%)	2 (3%)	0	0	0	0	77
27–28	67	25	2	0	0	0	94
weeks	(71%)	(27%)	(2%)				
34–35	16	54	38	12	0	0	120
weeks	(13%)	(45%)	(32%)	(10%)			
**Laughter AUs**							
24	78	38	4	0	0	0	120
weeks	(65%)	(32%)	(3%)				
27–28	57	43	8	0	0	0	108
weeks	(53%)	(40%)	(7%)				
34–35	21	53	31	8	1	0	113
weeks	(19%)	(47%)	(27%)	(7%)	(1%)		

Note: The “single” column indicate movement events for both fetuses where only a single AU is observed, the “double” column indicates events where two AUs occur together, up to the “sextuple” column, where six AUs co-occur.

**Figure 3:**
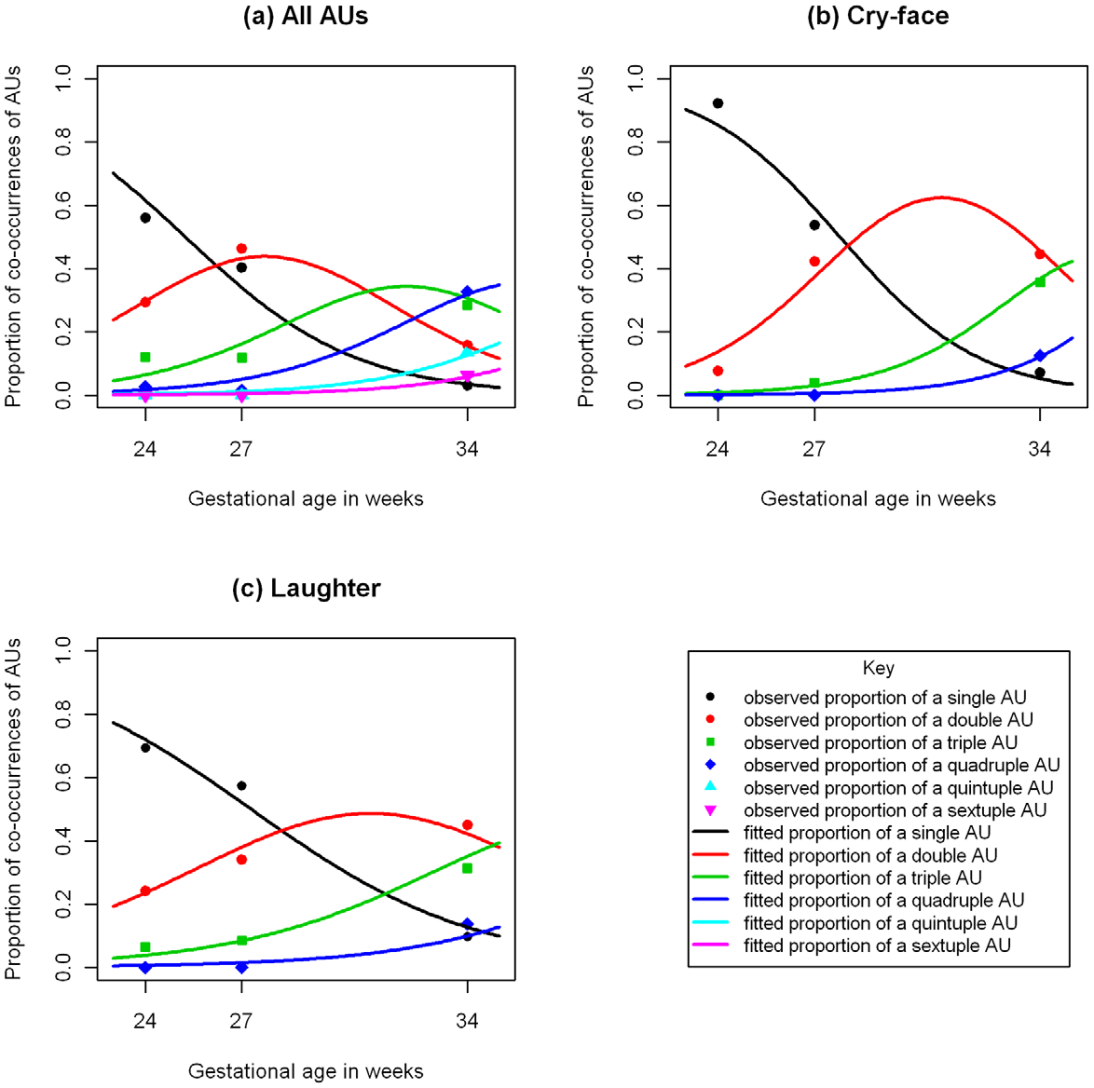
Graph showing increasing complexity of facial activity and expressions with gestational age for fetus A. In all displays, points show the observed proportions and lines show the fitted proportions from the ordinal regressions using linear age: a) Proportion of all Aus b) Proportion of AUs contributing to cry-face gestalt c) Proportion of AUs contributing to cry-face gestalt. In all three displays, the proportion of more complex combinations of AUs is increasing as the fetus ages.

Ordinal regression was carried out on each fetus separately, and showed that linear age (on the cumulative log-odds scale) was a significant predictor of increasing complexity with gestational age for both fetuses. (Fetus A: LRT = 137.4 on 1 df, p<0.001; Fetus B: LRT = 48.3 on 1 df, p<0.001). Figure 3a shows the fitted model for Fetus A. The fitted model for Fetus A identifies that the probability of a single AU declines to nearly zero by 34 weeks; the probability for a double AU increases and reaches a peak at 28 weeks before declining, and the probability of a triple AU reaches a peak at 32 weeks. Probabilities of observing quadruple, quintuple and sextuple AUS all continue to rise. The goodness of fit tests showed the linear age model fitted very well for fetus A (X^2^ =  9.5 on 9 df; p = 0.39) but less well for fetus B (X^2^ =  24.9 on 11 df; p = 0.01). The exponential age model for fetus B however fitted adequately (X^2^ =  19.4 on 1 df; p = 0.05).

For cry face gestalt AUs, ordinal regression showed that linear age was highly significant, with a progression showing increasing complexity over time (fetus A: LRT = 82.7 on 1 df, p<0.001; fetus B: LRT = 125.1 on 1 df, p<0.001). Figure 3b illustrates the nature of the developmental change for fetus A, with the fitted probabilities for triple and quadruple AUs increasing with age, and the probability of a single AU declining.

Similarly, for laughter gestalt AUs, ordinal regression showed that linear age was significant, with a progression showing increasing complexity over time (Fetus A: LRT = 54.3 on 1 df; p<0.001; Fetus B: LRT = 19.7 on 1 df; p<0.001). Figure 3c show very similar effects to Figure 3b: the probability of triple and quadruple AUs again increased with gestational age and that of single AUs again declined.

Both the cry-face and laughter gestalt models fitted well when assessed using the X^2^ statistic. For the cry gestalt, fetus A showed X^2^ = 2.4 on 5 df (p = 0.79) and fetus B showed X^2^ = 10.5 on 8 df:(p = 0.23). For the laughter gestalt, fetus A showed X^2^ = 3.5 on 5 df (p = 0.62) and fetus B showed X^2^ = 15.2 on 8 df (p = 0.06).

As the raw data in Table 2 gives the impression that the bulk of the change over time occurs in the third trimester, we also fitted the exponential time model to the cry-face and laughter gestalt data. For the cry-face gestalt, the exponential age model fitted less well than the linear age model for both fetuses: fetus A showed a Pearson goodness of fit test for the exponential age model of X^2^ = 12.1 on 5 df:p = 0.03 and fetus B showed X^2^ = 10.7 on 8 df: p = 0.22. For the laughter face gestalt the linear age model was preferred to the exponential age model for fetus A (exponential age model goodness of fit X^2^ = 4.25 on 5 df:p = 0.51); however, the exponential age model was preferred to the linear age model for fetus B (X^2^ = 9.06 on 8 df: p = 0.34). Thus, while the linear model fits well for both gestalts and both fetuses, for the laughter face gestalt for fetus B there is additional evidence that the exponential age model fits better. This demonstrates that for the laughter face gestalt the increasing trend to more complex facial movements is greater in the later gestational ages compared to the earlier ages for this fetus.

## Discussion

Based on the data of two fetuses, there is evidence to suggest that facial movements coalesce into specific facial gestalts. ‘Gestalts’ in the context of this paper refers to muscle configurations, rather than emotions usually defined to include subjective feeling, cognitive appraisal, physiological arousal and facial expression [30]. It is arguably possible to distinguish between volitional and emotional innervations of the face, which are controlled by different upper motor neurons [31]. Thus, facial displays in a social context may be regulated through cortical activity; whereas genuine emotional movements are not typically consciously controlled [31]. This might imply that emotions are present from birth or possibly pre-birth with neuronal development. However, although most emotional facial expressions are present very early in life, even simple emotions such as the social smile occur only after exposure to the social environment [21].

The present study confirms that over the second to third trimester, it is possible in the two fetuses studied to see a development from few facial action units observed in isolation at 24 weeks gestation to an impressive number of the 19 possible facial actions units observed at 34 to 35 weeks gestation Additionally the complexity of facial movements increases over time as indicated by the simultaneously expressed AUs forming facial gestalts. It has been argued and is physiologically consistent, that the non-vocal accompaniments of crying are developed before birth, but the vocal component is only functional at birth when the baby comes into contact with air [32]. The present findings support the concept that a fetus is capable of the complex motor behaviours that accompany the cry gestalt.

Given that the two fetuses show not only AUs common to both gestalts but also a combination of the AUs which distinguish laughter from cry, the findings suggest that around 34 weeks to 35 weeks gestation these gestalts are beginning to be established and differentiated from other gestalts. Especially, the cry face gestalt which was observed in the present study is consistent with the findings of others at 34 to 35 weeks [20]. Our findings underline the importance of examining individual facial muscle movements and relating each of these to gestalt content rather than making judgements based on overall impressions of observed facial movements, which may previously have led to inconsistencies and under-differentiated findings. In the present study we were able to show increasing complexity of fetal facial movement in two healthy female fetuses, over the second and third trimesters. Naturally, with only two fetuses, there are issues of representativeness, and future research is planned which will test these findings on a larger number of healthy male and female fetuses.

A separate issue concerns whether the developmental progression is linear on the log-odds scale or non-linear. The statistical analysis considered both forms of progression, and, in general, found that there was little evidence of non-linearity. The exception was for the laughter face gestalt for fetus B, where there was evidence of accelerating development over gestational age on the log-odds scale.

We have only considered two gestalts in this paper – laughter and cry. However, future work will consider other gestalts such as anger-face, smile-face and sadness-face. These will be defined by other combinations of the 19 AUs listed in Table 1.

Additionally, this foundational study suggests the considerable potential of this method for assessing fetal development. In future studies we intend to examine whether fetal facial movements might help differentiate between stressed and nonstressed fetuses. Previous research suggests that fetal behavioural patterns reflect the development of the fetal brain, it has been argued that ultrasound assessment of fetal behaviour can be used for the evaluation of the integrity of the fetal central nervous system (CNS) and, possibly, for the detection of functional or structural brain disorders (e.g., [33]).

Hence, subsequent discrimination of normal and abnormal fetal facial development might identify health problems in utero. Inevitably it will continue to offer new and fascinating insights into the remarkable process of fetal development.
